# A case of metastatic lymphoepithelial carcinoma of parotid gland identified on ^68^gallium DOTA-[Tyr3] octreotate PET CT

**DOI:** 10.1093/bjrcr/uaad011

**Published:** 2023-12-18

**Authors:** Han Chung Low, Kelvin Siu Hoong Loke, Fu Qiang Wang, Shuting Han, Amit Jain, Joe Yeong, Wen Long Nei

**Affiliations:** Department of Nuclear Medicine & Molecular Imaging, Singapore General Hospital, Singapore 169608, Singapore; Department of Nuclear Medicine & Molecular Imaging, Singapore General Hospital, Singapore 169608, Singapore; Division of Radiation Oncology, National Cancer Centre Singapore, Singapore 168583, Singapore; Division of Medical Oncology, National Cancer Centre Singapore, Singapore 168583, Singapore; Division of Medical Oncology, National Cancer Centre Singapore, Singapore 168583, Singapore; Institute of Molecular and Cell Biology (IMCB, A*STAR), Singapore 138673, Singapore; Department of Anatomical Pathology, Singapore General Hospital, Singapore 169608, Singapore; Division of Radiation Oncology, National Cancer Centre Singapore, Singapore 168583, Singapore

**Keywords:** lymphoepithelial carcinoma, EBV, somatostatin receptor, Ga-68 DOTATATE PET CT

## Abstract

The authors present the case of a 59-year-old lady diagnosed with lymphoepithelial carcinoma (LEC) of the left parotid gland. The primary tumour was identified using contrast-enhanced CT, and diagnosis was confirmed via fine needle aspiration cytology and immunohistochemistry. Staging using fluorine-18 fluorodeoxyglucose PET CT revealed regional nodal metastases, while no distant metastasis was evident. Following radical radiotherapy, a favourable locoregional response was observed on MRI, yet the patient's plasma Epstein-Barr virus load continued to rise. Given her primary tumour’s somatostatin receptor type 2 (SSTR2) positivity, gallium-68 DOTA-[Tyr3] octreotate PET CT (^68^Ga-DOTATATE PET CT) was performed, revealing multiple distant metastases with DOTATATE avidity. Despite attempts at palliative chemotherapy and immunotherapy, disease progression led to the decision for the best supportive care. The unique presentation of metastatic LEC on ^68^Ga-DOTATATE PET CT suggests a potential role for SSTR2-targeted imaging in diagnosis and management.

## Case presentation

Our patient was a 59-year-old lady with a history of hypertension and hyperlipidemia. She was a non-smoker who had a positive family history of breast cancer.

She presented with a painless left neck lump increasing in size over 3 months. She first noted it 2 weeks after her coronavirus disease-2019 vaccination and there were no other symptoms. Physical examination revealed a 6 cm firm, non-tender left parotid swelling. There was no facial nerve palsy.

## Investigations and imaging findings

**Figure 1.**
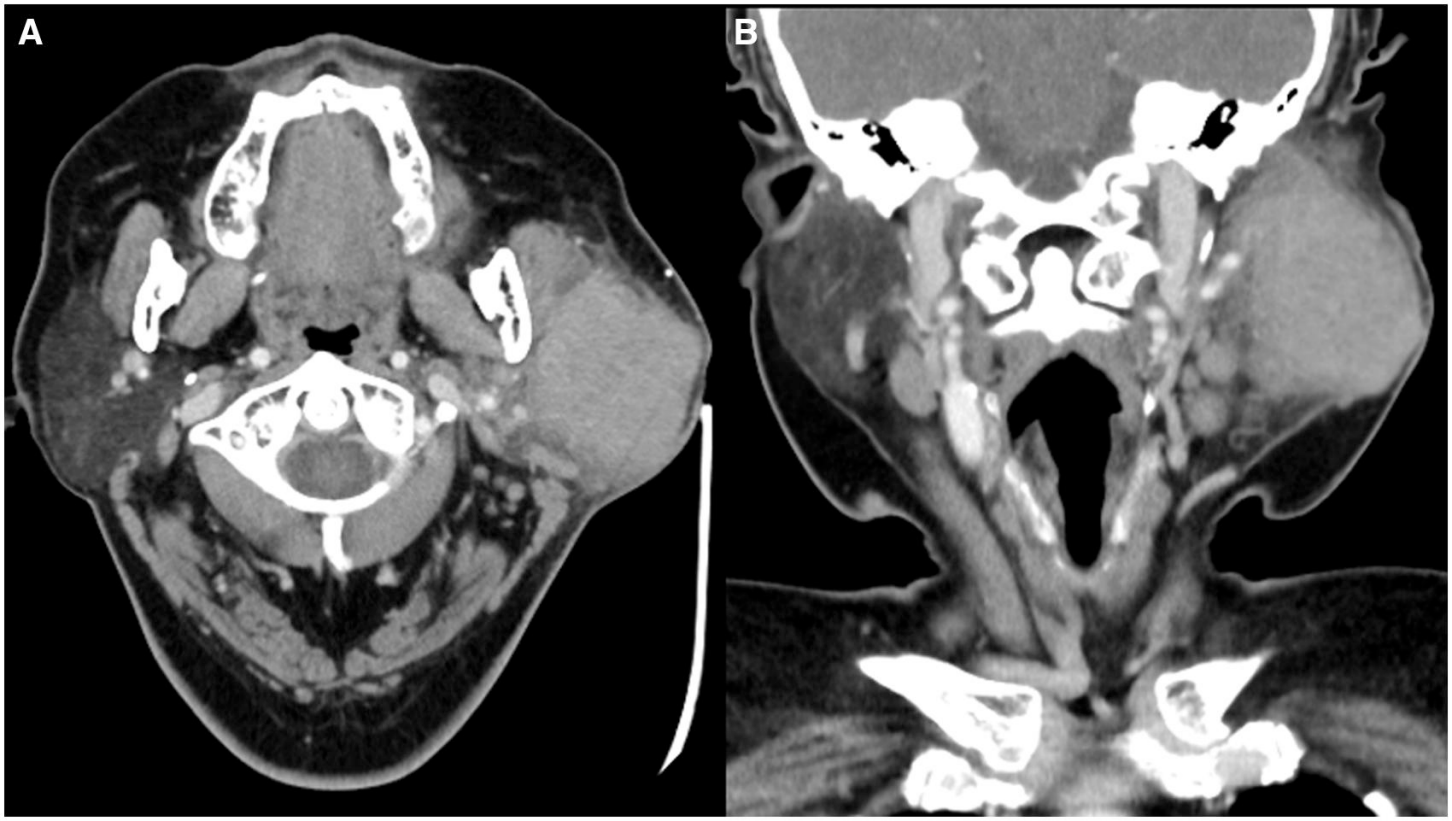
Contrast-enhanced CT ((A) axial; (B) coronal) shows an ill-defined enhancing lesion occupying almost the entire superficial left parotid gland with protrusion into the deep lobe.

A contrast-enhanced CT of the parotid glands was performed which revealed an ill-defined enhancing lesion occupying almost the entire superficial left parotid gland with protrusion into the deep lobe (Figure 1).

Fine needle aspiration of the lesion was performed, and cytology revealed lymphoepithelial carcinoma (LEC). Immunohistochemistry was positive for CK5-6, p40, and EBER-ISH, and negative for p16. The Ki-67/MIB-1 index was nearly 30%-50%. In view of this, the patient underwent post-nasal space biopsy to exclude a nasopharyngeal primary and no malignancy was detected.

**Figure 2.**
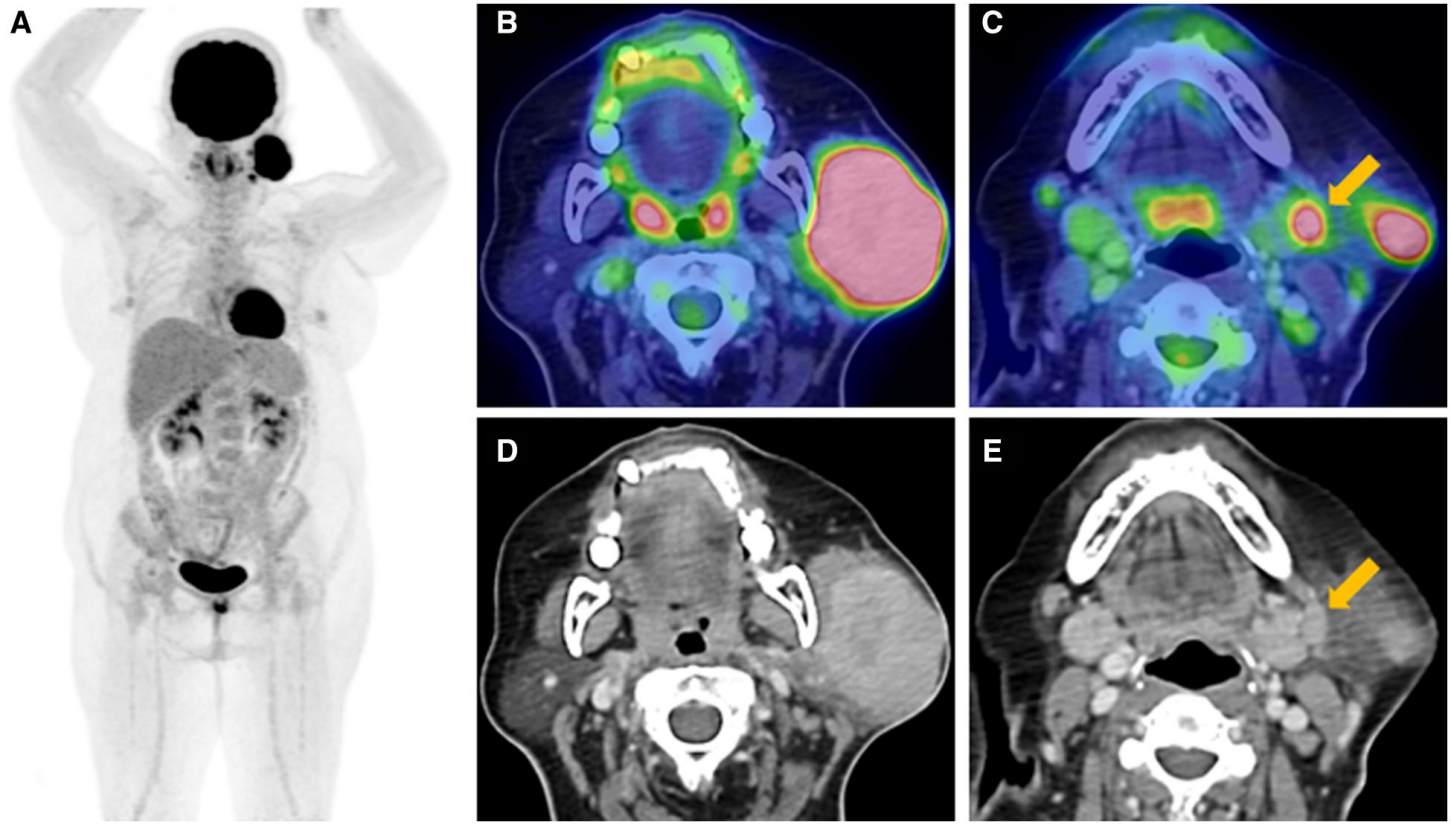
Fluorine-18 fluorodeoxyglucose PET CT ((A) maximum intensity projection; (B, C) axial hybrid; (D, E) axial CT) shows a hypermetabolic left parotid mass (B, D) and left cervical level Ib nodal metastases (C, E).

Fluorine-18 fluorodeoxyglucose PET CT (^18^F-FDG PET CT) was performed for initial staging and revealed a hypermetabolic left parotid mass and left cervical level Ib nodal metastases (Figure 2). No suspicious hypermetabolic lesion was seen in the post-nasal space and no hypermetabolic distant metastasis was detected.

## Treatment

The patient was offered surgery followed by adjuvant radiotherapy versus upfront radical radiotherapy with concurrent chemotherapy. She decided on upfront radical radiotherapy only and declined chemotherapy. She was given a radiation dose of 70 Gy divided into 35 fractions over 7 weeks.

## Outcome and follow-up

**Figure 3.**
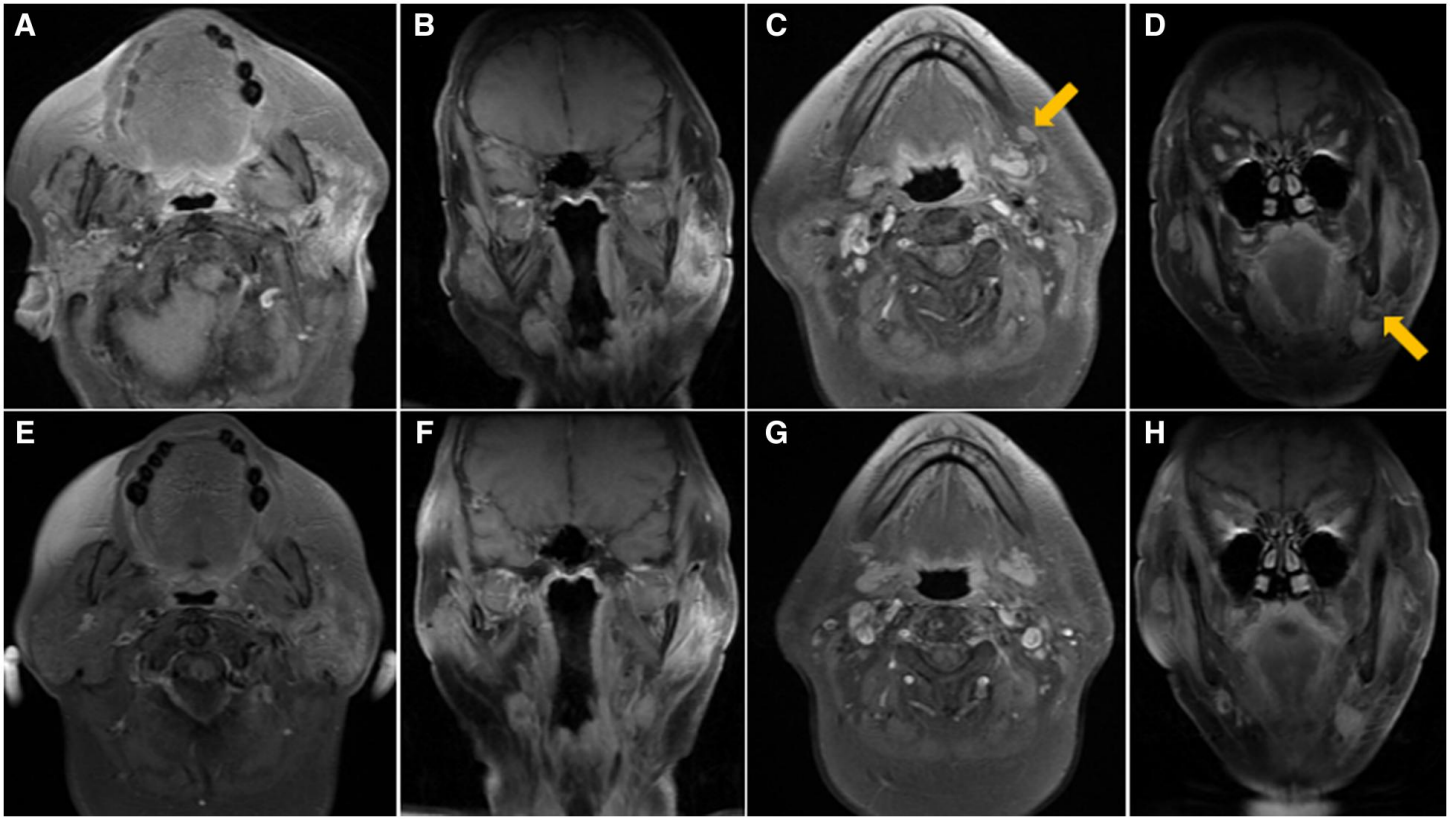
Post-contrast T1 weighted MRI with fat suppression performed 3 months (A-D) and 5 months (E-H) after completion of radiotherapy. First MRI shows significant decrease in size of the left parotid tumor ((A) axial, (B) coronal) and left cervical nodal metastases (arrows, (C) axial, (D) coronal). Second MRI shows further decrease in size of the left parotid tumour ((E) axial, (F) coronal) and resolution of the left cervical nodal metastases ((G) axial, (H) coronal).

Contrast-enhanced MRI of the neck was performed 3 months after completion of radiotherapy and showed significant decrease in size of the left parotid tumour and left cervical nodal metastases (Figure 3A-D). However, the patient’s plasma Epstein-Barr virus (EBV) DNA load had increased from less than 265 to 2079 copies/ml. MRI was repeated 2 months later and showed further decrease in size of the left parotid tumour and resolution of the left cervical nodal metastases (Figure 3E-H). However, the patient’s plasma EBV DNA load continued to increase to 31 625 copies/ml. The discordance between radiological findings and EBV DNA load raised the suspicion of metastatic disease.

**Figure 4.**
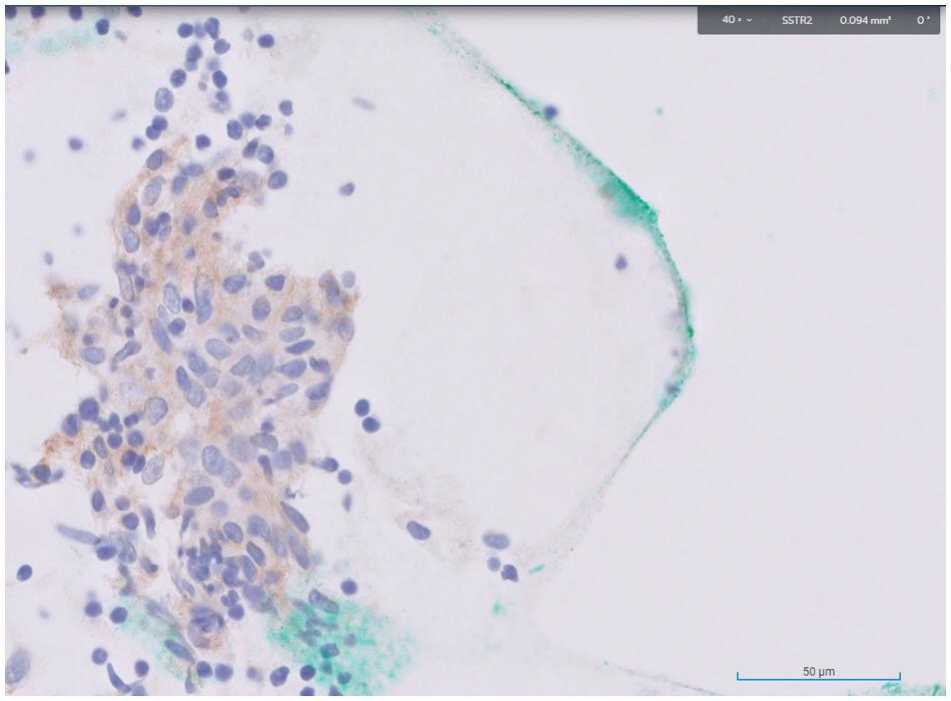
Immunohistochemical staining of the initial cytology specimen from the primary tumour showed expression of SSTR2 (stained brown) on the tumour cells (stained purplish blue).

The patient was recruited into an ongoing study on somatostatin receptor imaging in EBV-related cancers (NCT05581550) and further immunohistochemical staining of the initial cytology specimen showed the expression of somatostatin receptor type 2 (SSTR2) on the tumour cells (Figure 4).

**Figure 5.**
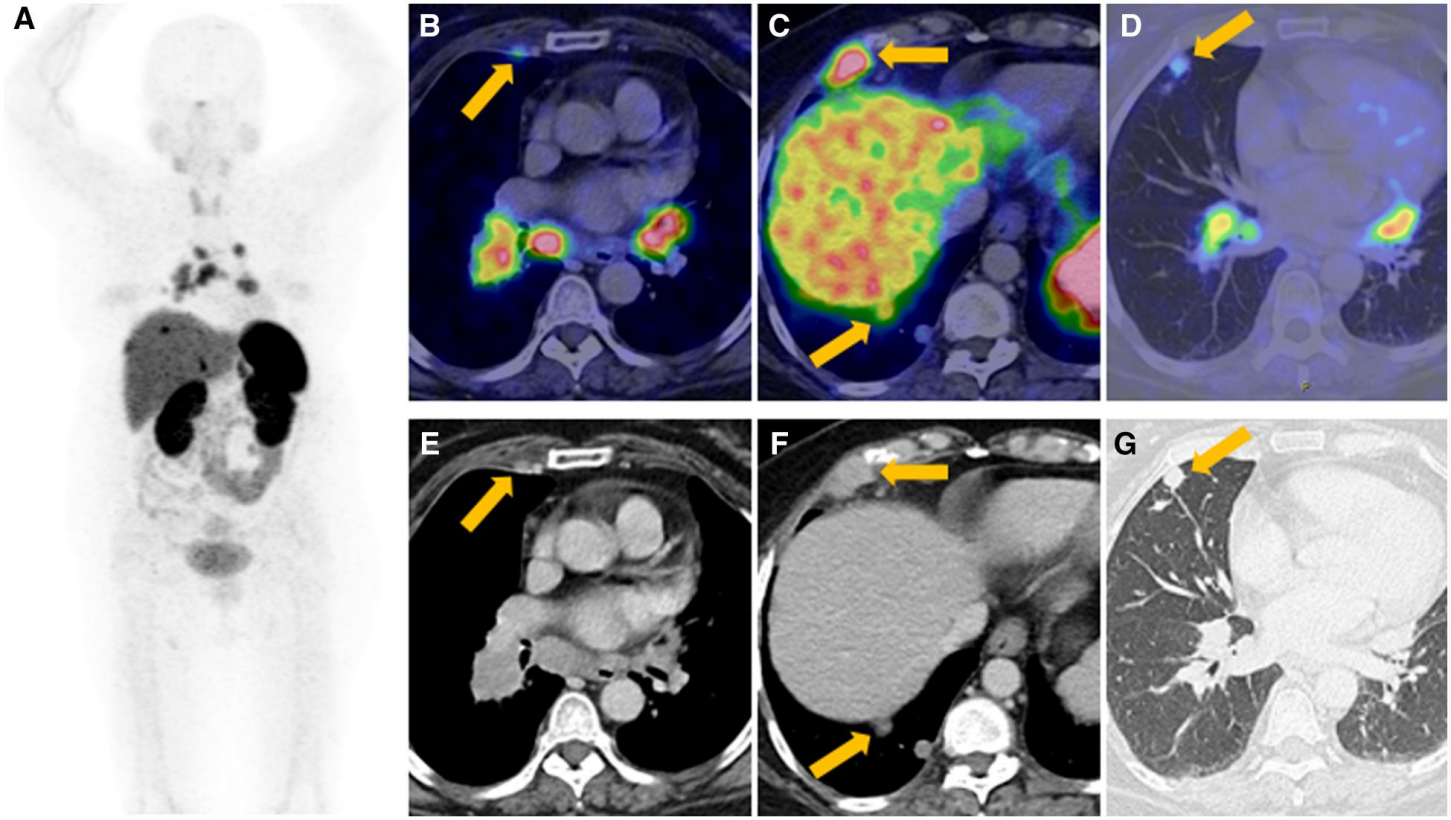
^68^Gallium DOTA-[Tyr3] octreotate PET CT ((A) maximum intensity projection; (B-D) axial hybrid; (E-G) axial CT) shows (B, E) multiple DOTATATE-avid mediastinal, bilateral interlobar and internal mammary (arrow) nodal metastases; (C, F) superior diaphragmatic and intercostal (upper arrow) nodal metastases; as well as pulmonary (arrow, D, G) and pleural (lower arrow, C, F) metastases.

The patient underwent gallium-68 DOTA-[Tyr3] octreotate PET CT (^68^Ga-DOTATATE PET CT) which revealed multiple new DOTATATE-avid mediastinal, internal mammary, bilateral interlobar, superior diaphragmatic, and intercostal nodal metastases, as well as multiple new non to mildly DOTATATE-avid bilateral pulmonary and right pleural metastases (Figure 5). There was interval decrease in size of the left parotid tumour which did not show significant DOTATATE uptake, in keeping with treated primary.

The patient was started on palliative chemotherapy with Gemcitabine and Carboplatin. Unfortunately, after two cycles of chemotherapy, she was hospitalized twice for pneumonia and chemotherapy had to be halted. She was not keen to restart chemotherapy after she recovered and opted to try immunotherapy. Following two cycles of Pembrolizumab, her disease continued to progress, and she was worse clinically. After discussion with the patient and her family, the decision for best supportive care was made.

## Discussion

Somatostatin, a hormone produced in the hypothalamus and gastrointestinal tract, exerts various neuroendocrine inhibitory effects in the body by binding to somatostatin receptors (SSTRs). Neuroendocrine neoplasms are rare and heterogeneous tumours primarily arising from the gastro-entero-pancreatic tract and lungs. They span a spectrum from well-differentiated neuroendocrine tumours (NETs) to poorly differentiated neuroendocrine carcinomas. Most NETs exhibit overexpression of SSTRs, with type 2 being the predominant subtype among the five. Somatostatin analogues (SSAs) such as octreotide and lanreotide are used to treat NETs by activating SSTRs to impede tumour growth and inhibit tumour-associated hormone secretion. SSTR expression on NETs can be imaged by labelling SSAs with a radionuclide, which was originally performed with ^111^In-pentetreotide (OctreoScan). Newer agents like DOTATATE, DOTATOC, and DOTANOC labelled with ^68^Ga have subsequently been developed for PET imaging. Since the mid-90s, SSAs labelled with ^177^Lu or ^90^Y have been used in peptide receptor radionuclide therapy for treating NETs.

Lymphoepithelial carcinomas are rare malignant tumours that are morphologically identical to undifferentiated nasopharyngeal carcinomas (NPC). LEC has been reported to arise primarily in multiple sites throughout the body except for the brain and central nervous system. In the head and neck, the salivary glands are the most common site of primary LEC after the nasopharynx. Like non-keratinizing NPC, primary LEC arising in the head and neck has a strong association with EBV particularly in EBV-endemic areas.^1^

Somatostatin receptor type 2 has been shown to be expressed in EBV-positive NPC.^2^ SSTR imaging with ^68^Ga-DOTATATE PET CT had been reported to detect primary and metastatic NPC.^2^^,^^3^

Our patient’s primary tumour was positive for EBV on immunohistochemistry and her plasma EBV DNA load was also detectable. Despite good locoregional response following treatment, her plasma EBV DNA load continued to increase, raising the suspicion of distant metastases. At this point, we decided to recruit her into an ongoing study on SSTR imaging in EBV related cancers. Initial specimen from her primary tumour was positive for SSTR2 on further immunohistochemistry and ^68^Ga-DOTATATE PET CT revealed multiple sites of DOTATATE-avid distant metastases.

To the best of our knowledge, this is the first report of metastatic LEC of parotid gland identified on ^68^Ga-DOTATATE PET CT. This case highlights the potential of SSTR2 as a molecular biomarker for PET CT imaging of patients with LEC of salivary glands. This may open new diagnostic and therapeutic options in the management of this disease.

## Learning points

- Like NPC, LEC of salivary glands has a strong association with EBV infection and serial monitoring of plasma EBV DNA load is useful for assessment of treatment response and detection of recurrence.
- EBV infection upregulates the expression of SSTR2 in NPC and may do likewise in LEC of salivary glands. It may be useful to perform immunohistochemical staining for SSTR2 on histology samples of LEC patients.
- SSTR2-targeted imaging such as ^68^Ga-DOTATATE PET CT may assist in the diagnosis, staging, and monitoring of patients with LEC, potentially leading to improved disease management.
